# Do Antidepressants Cure or Create Abnormal Brain States?

**DOI:** 10.1371/journal.pmed.0030240

**Published:** 2006-06-06

**Authors:** Joanna Moncrieff, David Cohen

## Abstract

Moncrieff and Cohen argue that psychotropic drugs create abnormal states that may co-incidentally relieve symptoms of mental illness.

The term antidepressant refers to a drug that helps to rectify specific biological abnormalities that give rise to the symptoms of depression. This exemplifies what we have called the “disease-centred” model of psychotropic drug action [ 1]. Modelled on paradigmatic situations in general medicine—such as the use of insulin in diabetes, antibiotics in infectious disease, chemotherapy in cancer—the disease-centred model suggests that antidepressants help restore normal functioning by acting on the neuropathology of depression or of depressive symptoms.

In contrast, we propose in this Essay that an alternative “drug-centred” model can better explain observed drug effects in psychiatric conditions. This drug-centred model suggests that instead of relieving a hypothetical biochemical abnormality, drugs themselves cause abnormal states, which may coincidentally relieve psychiatric symptoms ( Table 1). Alcohol's disinhibiting effects may relieve symptoms of social phobia, but that does not imply that alcohol corrects a chemical imbalance underlying social phobia. Sedation may lessen high arousal, present in many acute psychiatric situations. Drugs that induce indifference, such as neuroleptics or opiates, may help reduce the distress of acute psychotic symptoms. Low-dose stimulants may help improve attention and concentration in the short term.

**Table 1:**
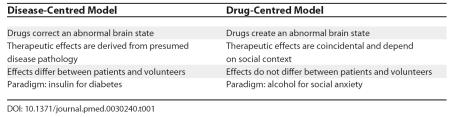
Main Assumptions of Two Models of Psychotropic Drug Action

The disease-centred model in psychiatry leads researchers to infer antidepressant effects from patients' scores on symptom rating scales presumed to assess the manifestations of the disease. The drug-centred model, on the other hand, suggests that physiological and subjective effects of drugs should be examined in their own right. These effects include various forms of sedation, stimulation, and a plethora of biopsychological states. Depending on individual inclination and context (including a person's emotional state upon drug ingestion), intoxication with some drugs produces euphoria or mood elevation. Because tolerance develops, however, euphoriant effects do not persist with long-term use. If antidepressants or any other psychotropic drugs could be shown to have mood-elevating effects that were long-term and not diminished by being in a depressed emotional state, this would distinguish them from psychotropic drugs that cause euphoria and might prove uniquely useful in depressed patients (see Sidebar).

## Evaluation of Alternative Models

The disease-based approach in psychiatry has rarely been tested directly. Prior to the dominance of this approach, which began in the 1960s, a drug-based model was mostly employed [ 1]. A disease-based model could be considered established if (1) the pathology of psychiatric conditions or symptoms had been delineated independently from the characterisation of drug action, and drug action could be extrapolated from that pathology; (2) rating scales used to evaluate drug treatment in clinical trials reliably detected changes in the manifestations of an underlying disease process rather than detecting drug-induced effects; (3) animal models of psychiatric conditions selected specific drugs; (4) drugs thought to have a specific action in certain conditions were shown to be superior to drugs thought to have nonspecific effects; (5) healthy volunteers showed different or absent patterns of effects, compared with diagnosed patients, since drugs would be expected to exert their therapeutic effects only in an abnormal nervous system [ 2]; and (6) the widespread use of supposedly disease-specific drugs led to demonstrable improvements in short- or long-term outcome of psychiatric disorders. Conversely, the absence of such evidence could indicate that a drug-centred model is preferable to guide scientific inquiry and produce therapeutic advances.

## Evidence for Disease-Based Action of Antidepressants

### The pathology of depression—the monoamine hypothesis

Antidepressants are believed to exert their therapeutic effects by acting on brain monoamines, which are believed to be important determinants of mood. However, in a circular chain of logic, the monoamine theory of depression was itself formulated primarily in response to observations that early antidepressants increased brain monoamine levels [ 3].

Independent evidence has not confirmed that there is a monoamine abnormality in depression. For example, the findings of brain imaging studies of serotonin abnormality are contradictory. Some found reduced serotonin 1A receptor binding in drug-free patients who were depressed, consistent with the hypothesis that selective serotonin reuptake inhibitors (SSRIs) improve depression by correcting a deficiency of serotonin activity [ 4, 5]. Other studies, however, have found no difference between patients who are drug-free and controls [ 6, 7] or increased binding potential in depressed patients [ 7, 8]. Postmortem findings of receptor changes in the brains of people who committed suicide have also been inconsistent [ 9–11]. In some studies, with patients who had recovered from depression, a tryptophan depletion challenge led to a transient increase in depressive symptoms. However, these results have not been confirmed in volunteer studies [ 12], and the effect appears to be dependent on previous SSRI use [ 13]. Research on catecholamines (noradrenaline and adrenalin) is similarly confusing and inconclusive [ 14].

### Depression rating scales

These scales contain items that are not specific to depression, including sleeping difficulties, anxiety, agitation, and somatic complaints. These symptoms are likely to respond to the nonspecific sedative effects that occur with most tricyclic antidepressants (TCAs) and some other antidepressants. Hence, changes in rating scale scores may merely reflect drug-induced effects.

### Animal models of depression

These models, which usually involve biochemical or behavioural processes thought to mimic aspects of depression in humans, do not select antidepressants reliably but produce numerous “false positives” with other drugs, including stimulants, opiates, and neuroleptics. They also produce some “false negatives” with supposed antidepressant drugs [ 15].

### Antidepressants versus other drugs

Many drugs not normally considered to be antidepressants show comparable effects to antidepressants when given to patients who are depressed in some randomised controlled trials (RCTs) [ 1, 16]. These include benzodiazepines [ 17], opiates [ 18], buspirone [ 19], stimulants [ 20], reserpine, and other antipsychotics [ 21].

### Healthy volunteer studies

The fact that antidepressants do not appear to elevate mood in healthy volunteers [ 22–26] might suggest that they have a disease-specific action. However, because of the nature of depression rating scales (as explained above), it is unclear that antidepressants specifically affect mood in patients who are depressed. Any effect they have over and above placebo may also be attributable to an “amplified” placebo response (see below). Although there are some reports of improved sleep in patients with depression who are given SSRIs versus volunteers' reports of decreased sleep when given SSRIs [ 27], in general, side effects in patient studies are consistent with effects on volunteers. For example, TCAs show sedation and cognitive impairment [ 28, 29], while SSRIs show gastrointestinal upset and drowsiness, both in patients and in healthy volunteers [ 22].

### Outcome of depression

There is little evidence outside RCTs that the long- or short-term outcome of depression is changing as a consequence of antidepressant use. Recent sharp increases in antidepressant use have been accompanied by increased prevalence and duration of depressive episodes [ 30] and rising levels of sickness absence [ 31]. Naturalistic studies have also shown that depressive episodes are more frequent and last longer among antidepressant users than among nonusers [ 30] and that sickness absence is more prolonged [ 32], although severity is likely to explain some of this effect (i.e., it is likely that patients are on antidepressant drugs because they have more severe disease). Follow-up studies of people treated for depression indicate high levels of nonrecovery or relapse [ 33–35].

## What Do Antidepressants Actually Do?

Since antidepressants come from a number of different chemical classes, they would be expected to produce different sorts of effects.

Most TCAs are strongly sedative and impair cognitive and motor performance [ 28, 29]. Amitriptyline causes profound electroencephalograph slowing similar to chlorpromazine [ 29]. Trazodone, mianserin, and mirtazapine also cause sedation and cognitive impairment [ 36, 37]. Research on SSRIs has found a “lack of profound effects in healthy subjects” (p. 17 of [ 22]). Studies with volunteers taking single doses show increased attention-test performance and motor speed, as well as sleep impairment, suggesting a slight stimulating effect [ 22, 27]. Studies with volunteers who have taken multiple doses over days or weeks show either no difference from placebo [ 37, 38] or impaired concentration, vigilance, and memory, and reports of drowsiness [ 22, 25, 39–41] compared with placebo, suggesting that SSRIs have mild sedative effects. Patient studies suggest that SSRIs may sometimes cause extreme and unpleasant activation or agitation [ 42–44], which can resemble neuroleptic-induced akathisia [ 45]. More commonly, SSRIs also cause subjective drowsiness or sedation [ 43]. It is therefore difficult to characterise overall effects of SSRIs, which may have simultaneous mild stimulant and sedating effects. Reboxetine appeared to be subjectively mildly stimulant or “energy enhancing” in one volunteer study [ 25].

In volunteer studies, measures of mood specifically address subjective feelings and show either no effects after antidepressant administration or dysphoria [ 22–26]. Two volunteer studies found slightly improved recognition of positive emotional material and reduced recognition of negative emotional states compared with placebo [ 23, 24]. Another found reduced reaction to negative events [ 26]. However, without a comparison with other drugs, one cannot know whether these are specific effects of the antidepressants tested, or simply consequences of an intoxicated state. Possibly, some antidepressants share the opiates' and neuroleptics' particular emotional blunting effects. Alternatively, drug-induced states may nonspecifically reduce emotional sensitivity.

## Drug Effects in Clinical Trials

RCTs of antidepressants report that drug-treated trial participants show greater improvement on rating scale scores than placebo-treated participants. However, this difference was shown to be small in recent meta-analyses—about two points on the Hamilton Rating Scale for Depression, or small differences in improvement rates [ 46, 47]. Drug-induced effects could account for this difference in several ways. In the Hamilton Rating Scale for Depression, for example, three items on sleep, two on anxiety and one on agitation can score up to 16 points (a total score between 19–22 on the Hamilton Rating Scale for Depression indicates severe depression). On these items, any drug with sedative effects would be likely to outperform placebo.

In addition, because inert placebos create nowhere near the range and intensity of effects (including side effects) that active drugs produce, RCTs of psychotropic drugs that use inert placebos (rather than active placebos, which mimic side effects of drugs) are not truly blinded [ 48]. In that case, outcomes for people on antidepressants are likely to be subject to amplified expectations compared with those on inert placebo [ 48]. This “placebo amplification” might be exacerbated in people who have taken antidepressants before and have not responded negatively [ 49]; modern trials are likely to select such patients above others [ 50, 51].

Therefore, RCT evidence cannot confirm that antidepressants have a specific mood-elevating effect in patients. This is consistent with evidence that they have no mood-elevating effect in volunteers. Drugs known to produce short-term euphoria require an increasing dose to maintain this effect (tolerance) and are associated with a compensatory dysphoria on discontinuation. Drugs such as antipsychotics cause dysphoria and some depressive symptoms [ 52]. So far, however, there is no compelling evidence that there exists any drug-induced effect consisting of a sustained elevation of mood. [Fig pmed-0030240-g001]

**Figure pmed-0030240-g001:**
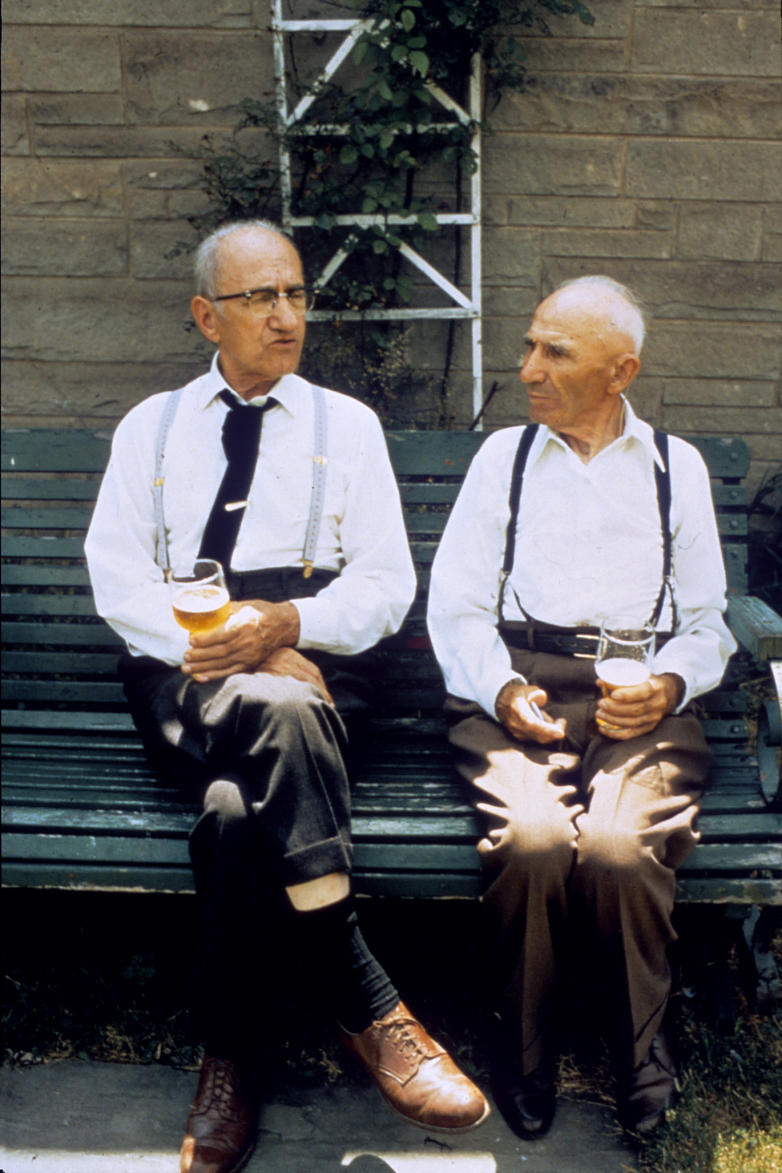
Alcohol can relieve symptoms of social phobia, but this does not mean that alcohol corrects a chemical imbalance underlying social phobia (Photo: J Troha/National Cancer Institute)

## Clinical and Theoretical Implications

The idea that antidepressant drugs target a specific biological state that produces depression strongly justifies the disease model of depression and its medical treatment [ 53]. Therefore, abandoning the disease-centred model of antidepressant action squarely challenges the notion of depression as a biologically based medical disease. The argument presented here supports claims that the medical concept of depression obscures the diversity of problems and experiences that come to be so labelled, and that social explanations and interventions have been undervalued [ 54, 55]. By contrast, a drug-centred model allows drug treatment to be considered without necessarily accepting a disease model.

A drug-centred model suggests that drug effects cannot easily be parcelled into “therapeutic” and “adverse” effects, since the same effect may have desirable and undesirable implications. Neuroleptic-induced indifference and sedation may help in acute psychosis but may impede long-term recovery. Increased passivity shown by a child on stimulants might help in a structured classroom but not in a summer camp. Drug use is always a fine balancing act, and patients' experiences are of primary importance in deciding whether there is more to be gained than lost. Such decisions require patients and professionals to cooperate to explore precisely what patients hope to achieve with drugs, matching these aims to known drug-induced effects.

Taking a drug-centred approach to the treatment of depression, we would conclude that no presently known effects of any drugs, including antidepressants, are likely to do more good than harm in the long term. In the short term, sedative effects of drugs may help people who are acutely anxious, highly aroused, or have difficulty sleeping. The common practice of prescribing short-term, low-dose sedative TCAs, for which general practitioners have frequently been criticised, may therefore be a rational one. Similarly, short-term benzodiazepine prescribing may occasionally be justified, bearing in mind the problem of dependency. On the other hand, although several drug classes (and possibly some antidepressants) are known to induce psychic indifference, the utility and desirability of this effect is doubtful.

## Conclusion

Many patients are led to believe, by their physicians and by advertising, that antidepressant drugs will act on the biological cause of their depressed state by rectifying a “chemical imbalance” [ 56]. On the contrary, our analysis indicates that there are no specific antidepressant drugs, that most of the short-term effects of antidepressants are shared by many other drugs, and that long-term drug treatment with antidepressants or any other drugs has not been shown to lead to long-term elevation of mood. We suggest that the term “antidepressant” should be abandoned. We have proposed an alternative drug-centred model of drug action that is consistent with a demedicalised approach to depression.

## Supporting Information

Text S1 Translation of Article Summary into French by David Cohen (38 KB DOC).

**Article Summary**

Antidepressants are assumed to work on the specific neurobiology of depressive disorders according to a “disease-centred” model of drug action. However, little evidence supports this idea. An alternative, “drug-centred,” model suggests that psychotropic drugs create abnormal states that may coincidentally relieve symptoms. Drug-induced effects of antidepressants vary widely according to their chemical class—from sedation and cognitive impairment to mild stimulation and occasionally frank agitation. Results of clinical trials may be explained by drug-induced effects and placebo amplification. No evidence shows that antidepressants or any other drugs produce long-term elevation of mood or other effects that are particularly useful in treating depression. (See Text S1 for French translation.)

## Abbreviations

RCT: randomised controlled trial
SSRI: selective serotonin reuptake inhibitor
TCA: tricyclic antidepressant
