# Supplementary material for: Do Antidepressants Cure or Create Abnormal Brain States?
Source: PLoS Med. 2006 Jun 6;3(7):e240. doi: 10.1371/journal.pmed.0030240 (PMC1472553; doi:10.1371/journal.pmed.0030240)
Supplement: Supplementary file 1 [file pmed.0030240.sd001.doc]

05-PLME-PF-0691R2

**Est-ce que les “antidépresseurs” soulagent ou provoquent des**

**dysfonctionnements cérébraux?**

Joanna Moncrieff & David Cohen

## *Résumé*

Selon un modèle d’action psychopharmacologique basé sur la maladie, les antidépresseurs agissent sur la neuropathologie des troubles dépressifs. Il existe cependant très peu d’évidence appuyant cette notion. Un modèle alternatif “centré sur le médicament” nous suggère que les substances psychotropes produisent des états cérébraux anormaux qui peuvent à l’occurence soulager divers symptômes. Selon leur classe chimique particulière, les antidépresseurs produisent une panoplie d’effets—incluant des effets sédatifs et dysfonctionnements cognitifs et des effets légèrement stimulants progressant parfois à l’agitation. Les résultats obtenus lors d’essais cliniques peuvent s’expliquer par divers effets médicamenteux et par un effet placébo amplifié. Mais il n’existe jusqu’à présent aucune évidence pour démontrer que les antidépresseurs ou toute autre substance produisent un rehaussement de l’humeur à long terme ou autre effet qui possède un avantage particulier dans le traitement de la dépression.
